# Predictors of outcomes in patients on peritoneal dialysis: A 2-year nationwide cohort study

**DOI:** 10.1038/s41598-019-40692-6

**Published:** 2019-03-08

**Authors:** Masanori Abe, Takayuki Hamano, Junichi Hoshino, Atsushi Wada, Shigeru Nakai, Norio Hanafusa, Ikuto Masakane, Kosaku Nitta, Hidetomo Nakamoto

**Affiliations:** 1 The Committee of Renal Data Registry, the Japanese Society for Dialysis Therapy, Tokyo, Japan; 2 Division of Nephrology, Hypertension and Endocrinology, Department of Internal Medicine, Nihon University School of Medicine, Tokyo, Japan; 3 Department of Inter-Organ Communication Research in Kidney Disease, Osaka University Graduate School of Medicine, Osaka, Japan; 4 Nephrology Center, Toranomon Hospital, Tokyo, Japan; 5 Department of Nephrology, Kitasaito Hospital, Asahikawa, Japan; 6 Department of Clinical Engineering, Fujita Health University, Aichi, Japan; 7 Department of Blood Purification, Tokyo Women’s Medical University, Tokyo, Japan; 8 Yabuki Hospital, Yamagata, Japan; 9 Department of Nephrology, Tokyo Women’s Medical University, Tokyo, Japan; 10 Department of General Internal Medicine, Saitama Medical University, Saitama, Japan

## Abstract

There has been no nationwide study of prognostic factors and outcomes in patients on peritoneal dialysis (PD) in Japan. We conducted a cohort study using data from the nationwide registry of the Japanese Society for Dialysis Therapy. We followed 8,954 prevalent PD patients for 2 years, 2014–2015. Cox proportional hazards regression analysis was used to determine factors that were independently associated with patient survival. Survival rates were compared between patients with and without diabetes after adjusting for potential confounders. During the 2-year study period, 893 (10.0%) of 8,954 patients died, 148 (1.6%) underwent kidney transplantation, and 2,637 (29.4%) were switched to hemodialysis; 5,276 (58.9%) patients were alive at the end of the study period. After multivariate adjustment, older age, longer duration of dialysis, presence of diabetes, cardiovascular comorbidity, use of 2.5% glucose dialysate, higher C-reactive protein and phosphate levels, and a lower serum albumin level were independently associated with increased hazard ratios for all-cause mortality. A combination of PD and hemodialysis was associated with a lower mortality rate. The new-onset cardiovascular event rate was significantly higher in the diabetes group than in the non-diabetes group (P < 0.0001). After adjusting for all variables, the hazard ratio was 1.509 (95% confidence interval 1.029–2.189, P = 0.036) in the diabetes group. Diabetes, older age, longer duration of dialysis, cardiovascular comorbidity, and inflammation were predictors of mortality in patients on PD.

## Introduction

In Japan, the limited availability of kidney transplantation as a form of renal replacement therapy means that most patients with end-stage kidney disease (ESKD) require lifelong dialysis. Therefore, the amount of time spent on hemodialysis (HD) or peritoneal dialysis (PD) is longer in Japan than in the US or European countries. The number of Japanese patients on long-term dialysis has been steadily increasing and reached 314,180 at the end of 2013, with 4.2% of these patients having been on dialysis for over 25 years^1^. Many studies have shown that patients on HD in Japan have lower rates of hospitalization and mortality than their counterparts in other countries^2–4^. However, there is limited information on hospitalization and mortality rates in patients on PD. Furthermore, the number of patients with diabetes-related ESKD continues to increase^5,6^, which means that the number of patients with diabetes on PD is also increasing^7^.

In view of the lack of data on outcomes in patients on PD in Japan, we undertook a cohort study using a nationwide registry of such patients to clarify the outcomes, including mortality and cardiovascular events, in patients with and without diabetes on PD.

## Methods

### Database creation

Data were obtained from annual nationwide surveys of patients on dialysis conducted by the Japanese Society for Dialysis Therapy (JSDT) and stored in the JSDT Renal Data Registry (JRDR). The surveys were conducted by volunteers, as described previously^7–10^. The study protocol was approved by the Medicine Ethics Committee of the Japanese Society for Dialysis Therapy and by our university. All procedures fully adhered to the Declaration of Helsinki. The study was registered with the University Hospital Medical Information Network (UMIN000018641). The need for informed consent was waived in view of the anonymity of the study data.

The study population comprised patients who were receiving PD on 31 December 2013 and were followed for 2 years. The inclusion criterion was PD for at least 90 days at the time of enrollment in the cohort. We excluded patients who had received only HD, those aged <20 years, those whose records for date of birth, initiation of dialysis, or outcome were incomplete, and those who were considered outliers. Patients were considered outliers if their anthropometric and laboratory measurements were outside the following ranges: height 120–200 cm, body weight 20–150 kg, serum albumin 1.0–5.0 g/dL, C-reactive protein (CRP) <30 mg/dL, and hemoglobin 5.0–20.0 g/dL. Demographic and clinical data at enrollment were collected, including age, sex, duration of dialysis, primary cause of ESKD, body mass index (BMI), smoking status, use of antihypertensive agents, and history of complications of cardiovascular disease (CVD), including cerebral infarction, cerebral hemorrhage, myocardial infarction, and limb amputation. The patients with diabetes in the study were those with a diagnosis of diabetic nephropathy as the primary cause of ESKD and those on antidiabetic medications. Blood samples were drawn and assayed at each dialysis center or hospital, and the most recent values, including serum albumin, hemoglobin, calcium, phosphate, intact parathyroid hormone (i-PTH), high-density lipoprotein (HDL) cholesterol, non-HDL cholesterol, and CRP at the time of survey were collected. Weekly Kt/V urea from residual renal function (renal Kt/V) and weekly Kt/V urea from PD (PD Kt/V) values were recorded. Total Kt/V was calculated as the sum of renal Kt/V and PD Kt/V. The ratio of creatinine in the dialysate to that in plasma (D/P Cr) was obtained using the peritoneal equilibration test. The type of PD fluid used, namely, 2.5% glucose or icodextrin dialysate, was recorded, since 1.5% glucose dialysate is used frequently in Japan. The use of an automated PD or connecting system was also recorded. The type of treatment was also recorded because combination PD + HD therapy, that is, HD on 1 day per week (performed in the same facility as PD) and PD on the other days, has been approved in Japan since 2010.

Cause of death was classified as CVD, infection, malignancy, gastrointestinal disease, encapsulating peritoneal sclerosis (EPS), hepatic disease, other, or unknown. We defined cardiovascular death as that due to chronic heart failure, pulmonary edema, ischemic heart disease (including acute myocardial infarction, i.e., death within 30 days of onset), arrhythmia, valvular heart disease, endocarditis, other cardiac disease, subarachnoid hemorrhage, intracerebral hemorrhage, cerebral infarction, or other brain disease. New-onset cardiovascular events, including cerebral infarction, cerebral hemorrhage, myocardial infarction, or limb amputation, and episodes of peritonitis were also recorded. The definition of peritonitis used was that in the International Society for Peritoneal Dialysis guidelines^11^. The dates and causes of death were obtained from the JRDR at the end of 2014 and 2015.

### Outcome analysis by demographic factors, dialysis dose, and nutritional factors

We divided the patients into six a priori categories based on duration of PD (<2, 2 to <4, 4 to <6, 6 to <8, 8 to <10, and ≥10 years) to examine the relationship between category of dialysis duration and risk of death. The patients were also categorized by dialysis-related factors, including residual renal function, i.e., anuric or non-anuric state, use of 2.5% glucose or icodextrin dialysate, and use of an automated PD or connecting system. We also divided the patients into five a priori categories based on total Kt/V, i.e., <1.1 and ≥2.0, with intervening increments of 0.3, to examine the dose-response association between the categories of Kt/V and risk of death. BMI, hemoglobin, albumin, non-HDL cholesterol, HDL cholesterol, calcium, phosphate, and CRP levels were included as nutrition-related and inflammation-related factors. Age, hemoglobin, CRP, BMI, and serum albumin, non-HDL cholesterol, HDL cholesterol, calcium, and phosphate levels were analyzed as continuous variables. We estimated the association between demographic factors at enrollment and survival using multivariate Cox proportional hazards regression and analyzed models that were both unadjusted and adjusted for relevant covariates measured at study enrollment, including age, sex, duration of PD, presence or absence of diabetes, comorbid CVD, use of antihypertensive agents, residual renal function, total Kt/V, type of dialysate, hemoglobin, CRP, BMI, albumin, non-HDL cholesterol, HDL cholesterol, calcium, and phosphate. Patients who were switched to HD or underwent kidney transplantation during follow-up were censored from the analysis of outcomes.

### Outcome analysis by diabetes and non-diabetes groups

We divided the patients into two groups based on the presence or absence of diabetes. Survival analyses with Cox proportional hazards regression were used to examine whether the presence or absence of diabetes predicted survival for up to 2 years of follow-up. Kaplan-Meier survival analysis was used to compare all-cause, cardiovascular, and infection-related mortality rates. The log-rank statistic was used to test for differences between the two groups. New-onset cardiovascular events were also analyzed. Finally, we examined associations between the two groups and all-cause mortality. Models were analyzed when adjusted for the above-mentioned demographic, dialysis-related, nutrition-related, and inflammation-related factors measured at enrollment.

### Statistical analysis

Data were summarized using proportions, with mean ± standard deviation or median (interquartile range) as appropriate. Categorical variables were analyzed using the chi-square test and continuous variables using the Student’s *t*-test. Categorical data were compared between groups using repeated-measures analysis of variance and Tukey’s honestly significant difference test or Kruskal-Wallis test as appropriate. Missing covariate data were imputed by the conventional method for multivariate regression as appropriate. Proportional hazards assumption was tested by graphical and formal testing. All analyses were performed using JMP® version 13.0 (SAS Institute, Cary, NC). A P-value < 0.05 was considered statistically significant.

## Results

### Patient demographics and clinical characteristics

**Figure 1:**
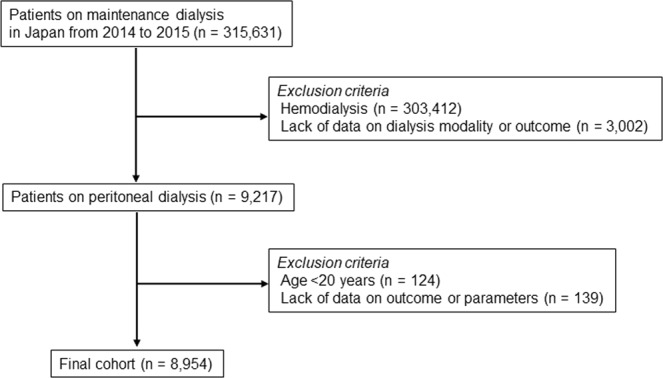
Flowchart showing enrolment of participants.

In total, 315,631 patients on maintenance dialysis were registered at the end of 2013. After exclusions, 8,954 patients on PD were enrolled in the study (Fig. 1). Demographic and clinical characteristics of the 8,954 patients at enrollment are shown in Table 1. Mean patient age was 62.7 ± 13.1 years, 63.8% of the patients were male, and 15.1% had comorbid CVD, comprising coronary artery disease, stroke, or limb amputation. Mean duration of PD was 33 (15–60) months, mean BMI was 23.4 ± 3.8 kg/m^2^, and mean albumin and hemoglobin levels were 3.3 ± 0.5 g/dL and 10.8 ± 1.4 g/dL, respectively. The most common cause of ESKD was glomerulonephritis (39.2%), followed by diabetic nephropathy (30.3%) and hypertension (12.9%). Renal Kt/V and PD Kt/V values at enrollment were 0.3 (0–0.8) and 1.4 ± 0.6, respectively. An automated PD system was used in 43.2% of patients and a connecting device in 68.2%. During the 2-year study period, 893 patients (10.0%) died, 148 (1.6%) underwent kidney transplantation, 2,637 (29.4%) were switched to HD; 5,276 (58.9%) patients were still alive at the end of the study period.

**Table 1:** Demographic, clinical, and laboratory data at baseline in 8,954 patients on peritoneal dialysis.

Variable	
Patients (n)	8,954
Age (years)	62.7 ± 13.1
Male sex (%)	63.8
Dialysis duration (months)	33 [15–60]
Primary cause of ESKD, n (%)	
Chronic glomerulonephritis	3,513 (39.2)
Diabetic nephropathy	2,710 (30.3)
Hypertension	1,154 (12.9)
Polycystic kidney disease	223 (2.5)
Other	476 (5.3)
Unknown	878 (9.8)
Body mass index (kg/m^2^)	23.4 ± 3.8
Smoking (%)	8.1
Antihypertensive agents (%)	79.2
Comorbid CVD (%)	15.1
Hemoglobin (g/dL)	10.8 ± 1.4
Albumin (g/dL)	3.3 ± 0.5
Non-HDL cholesterol (mg/dL)	128 ± 37
HDL cholesterol (mg/dL)	50 ± 18
C-reactive protein (mg/dL)	0.13 [0.07–0.40]
Calcium (mg/dL)	8.7 ± 0.8
Phosphate (mg/dL)	5.2 ± 1.3
Intact PTH (pg/mL)	155 [81–262]
Renal Kt/V	0.3 [0–0.8]
PD Kt/V	1.4 ± 0.6
Total Kt/V	1.8 ± 0.8
D/P Cr	0.65 ± 0.13
Use of 2.5% glucose dialysate (%)	37.5
Use of icodextrin (%)	40.8
Use of automated PD (%)	43.2
Connecting system (%)	
Device (+)	68.2
PD + HD combination (%)	18.8

Data are presented as the number, percentage, mean ± standard deviation, or median [interquartile range]. Cr, creatinine; CVD, cardiovascular disease; D/P, dialysate/plasma; ESKD, end-stage kidney disease; HD, hemodialysis; HDL, high-density lipoprotein; PD, peritoneal dialysis; PTH, parathyroid hormone.

### All-cause mortality according to demographic, dialysis-related, nutrition-related, and inflammation-related factors at enrollment

Hazard ratios (HRs) in the unadjusted analysis of variables evaluated as potential predictors of mortality in all patients are presented in Table 2. There was no significant effect of sex on patient survival (P = 0.114). Older age, longer duration of PD, presence of diabetes, and comorbid CVD were significant predictors of mortality. Lower dialysis dose, assessed by total Kt/V, was associated with higher risk of mortality. Use of 2.5% glucose or icodextrin dialysate and an anuric state at enrollment were also significant predictors of mortality. Poor nutritional status and more severe inflammatory status, indicated by lower hemoglobin, higher CRP, lower BMI, lower serum albumin, lower calcium, and lower phosphate, were also associated with higher mortality in patients on PD. PD + HD combination therapy was associated with a lower mortality risk. After multivariate adjustment, older age, longer duration of PD, presence of diabetes, comorbid CVD, use of 2.5% glucose dialysate, higher CRP, lower BMI, and lower albumin and phosphate levels continued to be independently associated with increased HR for all-cause mortality (Table 2). Use of antihypertensive agents, higher HDL levels, and PD + HD combination therapy continued to be associated with lower mortality risk.

**Table 2:** Hazard ratios (with 95% CIs) for variables evaluated as potential predictors of all-cause mortality in the study population.

Factors	n (%)	Unadjusted			Adjusted		
		HR	95% CI	P-value	HR	95% CI	P-value
Sex							
Male	5,714 (63.8)	1.000	Reference	Reference	1.000	Reference	Reference
Female	3,240 (36.2)	0.898	0.787–1.025	0.114	0.772	0.438–1.378	0.376
Age							
1-year increase	8,954 (100)	1.081	1.073–1.087	<0.0001	1.113	1.078–1.149	<0.0001
PD duration (years)							
<2	3,507 (39.2)	1.000	Reference	Reference	1.000	Reference	Reference
≥2 <4	2,344 (26.2)	1.170	0.978–1.399	0.086	1.374	0.966–1.953	0.076
≥4 <6	1,422 (15.9)	1.432	1.165–1.762	0.001	1.627	1.173–2.256	0.004
≥6 <8	761 (8.5)	1.676	1.233–2.278	0.001	1.720	1.041–2.841	0.045
≥8 <10	414 (4.6)	1.545	1.093–2.183	0.013	2.211	1.377–3.549	0.002
≥10	506 (5.6)	1.506	1.113–2.036	0.007	2.979	1.687–5.259	0.002
Diabetes							
No	5,635 (62.9)	1.000	Reference	Reference	1.000	Reference	Reference
Yes	3,319 (37.1)	1.500	1.318–1.708	<0.0001	1.648	1.097–2.474	0.016
Comorbid CVD							
No	7,399 (84.9)	1.000	Reference	Reference	1.000	Reference	Reference
Yes	1,313 (15.1)	2.861	2.464–3.322	<0.0001	1.367	1.055–1.771	0.017
Use of antihypertensive medication							
No	1,264 (20.8)	1.000	Reference	Reference	1.000	Reference	Reference
Yes	4,802 (79.2)	0.509	0.422–0.615	<0.0001	0.667	0.501–0.889	0.005
Residual renal function							
Non-anuric	3,479 (77.2)	1.000	Reference	Reference	1.000	Reference	Reference
Anuric	1,026 (22.8)	1.677	1.330–2.114	<0.0001	1.257	0.785–1.984	0.335
Total Kt/V							
<1.1	286 (8.7)	2.313	1.481–3.614	0.001	2.431	0.925–6.386	0.071
≥1.1 <1.4	326 (9.9)	1.392	0.806–2.405	0.235	1.162	0.419–3.222	0.772
≥1.4 <1.7	682 (20.7)	1.000	Reference	Reference	1.000	Reference	Reference
≥1.7 <2.0	853 (25.9)	1.159	0.784–1.713	0.457	0.792	0.350–1.791	0.576
≥2.0	1,144 (34.8)	0.832	0.563–1.230	0.358	0.614	0.262–1.437	0.262
Use of 2.5% dialysate							
No	3,422 (62.5)	1.000	Reference	Reference	1.000	Reference	Reference
Yes	2,056 (37.5)	1.502	1.267–1.779	<0.0001	2.551	1.433–4.539	0.001
Use of icodextrin dialysate							
No	3,241 (59.2)	1.000	Reference	Reference	1.000	Reference	Reference
Yes	2,237 (40.8)	1.307	1.102–1.548	0.002	0.968	0.641–1.445	0.876
PD + HD combination therapy							
No	7,275 (81.2)	1.000	Reference	Reference	1.000	Reference	Reference
Yes	1,679 (18.8)	0.556	0.438–0.703	<0.0001	0.595	0.413–0.858	0.005
Hemoglobin							
1-g/dL increase	6,130 (68.5)	0.773	0.726–0.823	<0.0001	0.925	0.844–1.014	0.098
C-reactive protein							
1-mg/dL increase	5,344 (59.7)	1.571	1.467–1.682	<0.0001	1.217	1.073–1.381	0.002
Body mass index							
1-kg/m^2^ increase	5,330 (59.5)	0.910	0.885–0.935	<0.0001	0.906	0.847–0.969	0.004
Serum albumin							
1-g/dL increase	6,092 (68.0)	0.217	0.190–0.247	<0.0001	0.363	0.234–0.562	<0.0001
Non-HDL cholesterol							
1-mg/dL increase	3,658 (40.9)	0.996	0.993–0.999	0.042	0.995	0.989–1.001	0.097
HDL cholesterol							
1-mg/dL increase	4,264 (47.6)	0.982	0.976–0.989	<0.0001	0.981	0.968–0.995	0.008
Calcium (mg/dL)							
1-mg/dL increase	6,175 (69.0)	0.748	0.685–0.819	<0.0001	1.145	0.877–1.493	0.317
Phosphate (mg/dL)							
1-mg/dL increase	7,340 (82.0)	0.871	0.818–0.925	<0.0001	1.231	1.046–1.450	0.012

CI, confidence interval; CVD, cardiovascular disease; HD, hemodialysis; HDL, high-density lipoprotein; HR, hazard ratio; PD, peritoneal dialysis.

In total, 1,240 episodes of PD-related peritonitis occurred in 859 patients during the study period. The mean peritonitis rate was 0.18 per patient-year. Multivariate analysis was performed to identify predictors of peritonitis (Supplementary Table 1). A previous history of peritonitis, use of a connecting device, comorbid CVD, and higher BMI were independent predictors of peritonitis.

### Demographics and clinical characteristics in the diabetes and non-diabetes groups

Patients were divided into two groups based on the presence or absence of diabetes. Table 3 shows the demographics and clinical characteristics of each group. Patients with diabetes were older and more likely to be male, with shorter duration of PD, higher BMI, higher rate of CVD comorbidity, lower HDL cholesterol, and higher CRP levels. There was no significant difference in hemoglobin level, total Kt/V value, or frequency of use of an automated PD system. More patients in the diabetes group used 2.5% glucose and/or icodextrin dialysate than those in the non-diabetes group.

**Table 3:** Demographic, clinical, and laboratory data in diabetes and non-diabetes patients on peritoneal dialysis.

	Diabetes group	Non-diabetes group	P-value
Patients (n)	3,319	5,635	—
Age (years)	63.9 ± 11.8	61.9 ± 13.6	<0.0001
Male sex (%)	70.4	59.9	<0.0001
PD duration (m)	26 [13–48]	37 [17–69]	<0.0001
Type 1 diabetes (%)	8.3	—	—
Body mass index (kg/m^2^)	24.3 ± 3.8	22.7 ± 3.6	<0.0001
Smoking (%)	9.9	6.9	<0.0001
Antihypertensive agents (%)	81.7	77.5	<0.0001
Comorbid CVD (%)	21.4	11.3	<0.0001
Albumin (g/dL)	3.2 ± 0.5	3.3 ± 0.5	<0.0001
Non-HDL cholesterol (mg/dL)	126 ± 38	130 ± 36	0.004
HDL cholesterol (mg/dL)	46 ± 16	52 ± 17	<0.0001
HbA_1c_ (%)	6.2 ± 1.1	—	—
C-reactive protein (mg/dL)	0.17 [0.09–0.49]	0.11 [0.06–0.37]	0.001
Calcium (mg/dL)	8.6 ± 0.8	8.8 ± 0.8	<0.0001
Phosphate (mg/dL)	5.2 ± 1.3	5.3 ± 1.3	0.028
Intact PTH (pg/mL)	150 [77–249]	158 [82–267]	0.002
Hemoglobin (g/dL)	10.8 ± 1.4	10.8 ± 1.4	0.313
Renal Kt/V	0.4 [0–0.9]	0.2 [0–0.7]	<0.0001
PD Kt/V	1.3 ± 0.6	1.4 ± 0.6	<0.0001
Total Kt/V	1.8 ± 0.8	1.8 ± 0.8	0.848
D/P Cr	0.67 ± 0.13	0.64 ± 0.13	<0.0001
PET category (%)			<0.0001
Low	8.1	11.6	
Low average	36.7	39.1	
High average	42.1	40.5	
High	13.1	8.8	
Use of 2.5% glucose dialysate (%)	40.1	36.0	0.002
Use of icodextrin (%)	45.0	38.3	<0.0001
Use of APD system (%)	43.3	43.1	0.924
Connecting system (%)			
Device (+)	72.5	65.5	<0.001
PD + HD combination (%)	18.5	19.0	0.537

Data are shown as the number, percentage, mean ± standard deviation, or median [interquartile range]. APD, automated peritoneal dialysis; Cr, creatinine; CVD, cardiovascular disease; D/P, dialysate/plasma; HD, hemodialysis; HDL, high-density lipoprotein; PD, peritoneal dialysis; PET, peritoneal equilibration test; PTH, parathyroid hormone.

### Comparison of outcomes between the diabetes and non-diabetes groups

**Figure 2:**
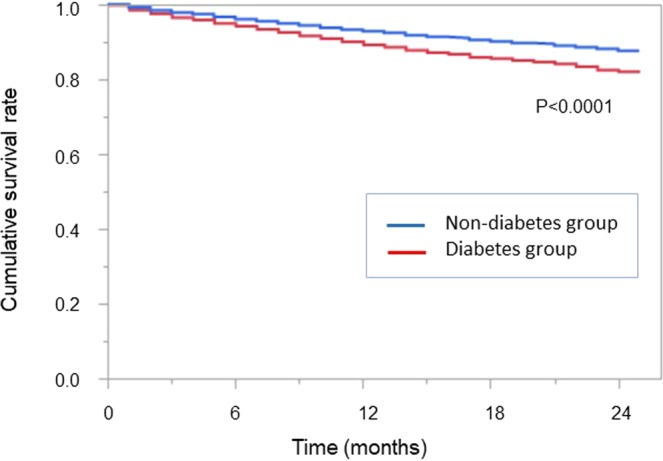
Comparison of cumulative survival rates between diabetes and non-diabetes patients on peritoneal dialysis.

Outcome variables are shown in Table 4. In total, 1,020 cardiovascular events (401 fatal, 619 non-fatal) were recorded during the study period. The cardiovascular event rate was significantly higher in the diabetes group than in the non-diabetes group (15.9% vs. 8.7%; P < 0.0001). The leading cause of death in the diabetes group was CVD (42.1%), followed by infection (27.2%). The leading cause of death in the non-diabetes group was also CVD (35.7%), followed by infection (21.7%). EPS was the cause of death in 1 patient in the diabetes group and in 5 patients in the non-diabetes group; the difference was statistically significant (P = 0.0013). Kaplan-Meier survival analysis (Fig. 2) showed a 2-year survival rate of 82.2% in the diabetes group and 87.4% in the non-diabetes group (P < 0.0001). Kaplan-Meier survival analyses for cardiovascular and infection-related deaths revealed significant differences in the patient survival rates between the diabetes and non-diabetes groups (both P < 0.0001; Supplementary Fig. 2a,b).

**Table 4:** New-onset cardiovascular event rates and causes of death in diabetes and non-diabetes patients.

	All	Diabetes group	Non-diabetes group	P-value^a^
New-onset CV events, n (%)	1,020 (11.4)	527 (15.9)	493 (8.7)	<0.0001
Death, n (%)	893 (14.5)	397 (17.8)	496 (12.7)	<0.0001
Cause of death, n (%)				0.001
Cardiovascular disease	344 (38.5)	167 (42.0)	177 (35.7)	
Infection	215 (24.1)	108 (27.2)	107 (21.6)	
Malignancy	50 (5.6)	13 (3.3)	37 (7.5)	
Gastrointestinal disease	29 (3.2)	7 (1.8)	22 (4.4)	
EPS	6 (0.7)	1 (0.3)	5 (1.0)	
Hepatic disease	12 (1.3)	6 (1.5)	6 (1.2)	
Other	130 (14.6)	48 (12.1)	82 (16.5)	
Unknown	107 (12.0)	47 (11.8)	60 (12.1)	

^a^Diabetes vs. non-diabetes. CV, cardiovascular; EPS, encapsulating peritoneal sclerosis.

### HRs for all-cause mortality between the diabetes and non-diabetes groups

**Figure 3:**
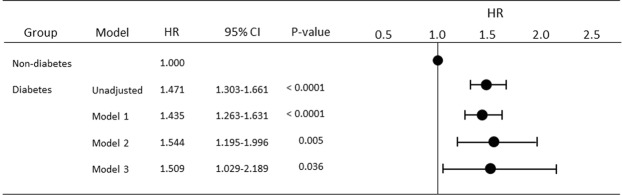
Comparison of hazard ratios for all-cause mortality between diabetes and non-diabetes patients on peritoneal dialysis using standard Cox proportional hazards regression. Model 1 is adjusted for age, sex, duration of PD, and presence or absence of comorbid cardiovascular disease. Model 2 is adjusted for basic demographic and dialysis-related factors, including total Kt/V, anuric or non-anuric state, type of dialysate, and PD + HD combination therapy. Model 3 is adjusted for demographic, dialysis-related, nutrition-related, and inflammation-related factors, including body mass index and C-reactive protein, hemoglobin, serum albumin, non-HDL cholesterol, HDL cholesterol, calcium, and phosphate levels. CI, confidence interval; HD, hemodialysis; HDL, high-density lipoprotein; HR, hazard ratio; PD, peritoneal dialysis.

As shown in Fig. 3, unadjusted HR for all-cause mortality in the diabetes group when compared with that in the non-diabetes (reference) group was 1.471 (95% CI 1.303–1.661; P < 0.0001). After adjusting for sex, age, duration of PD, smoking status, and comorbid CVD, HR for all-cause mortality in the diabetes group when compared with the non-diabetes (reference) group was 1.435 (95% CI 1.263–1.631; P < 0.0001). After adjusting for modification of dialysis, including total Kt/V value, anuric state, use of 2.5% glucose dialysate or icodextrin, and PD + HD combination therapy, as well as for baseline factors, HR for the diabetes group compared with the non-diabetes (reference) group was 1.544 (95% CI 1.195–1.996; P = 0.005). Finally, after adjusting for nutrition-related and inflammation-related factors in addition to the demographic factors and dialysis-related factors, the HR for the diabetes group when compared with the non-diabetes (reference) group was 1.509 (95% CI 1.029–2.189; P = 0.036).

## Discussion

This study is the first to identify predictors of 2-year mortality in patients on PD in Japan. Duration of PD and the serum albumin level were the most common factors associated with patient survival. Interestingly, PD + HD combination therapy was associated with lower mortality risk. The patients with diabetes who were receiving PD had lower survival rates than those without diabetes. The main strength of this study is that it is a nationwide cohort analysis of outcomes in patients on PD in Japan. Moreover, this is the first Japanese study to address prognostic risk factors in these patients.

Data from the US Renal Data System for 2010 revealed that the overall 1-year, 2-year, and 3-year survival rates of patients on PD were 83%, 67.2%, and 53.8%, respectively, whereas those in a diabetes subgroup were 80.3%, 61.7%, and 46.6%^12^. A further study from Canada reported 1-year, 2-year, and 3-year survival rates of 91%, 76%, and 66%, respectively, for patients with diabetes and respective rates of 94%, 89%, and 84% for patients without diabetes^13^. Data from single centers in Asia and Europe also showed that survival rates were significantly lower in patients with diabetes on PD than in their counterparts without diabetes^14^. In the present study, the survival rate was significantly lower in the diabetes group than in the non-diabetes group. However, the 1-year and 2-year survival rates in all patients in our study were 91.4% and 85.5%, respectively, and higher than in previous reports; furthermore, the respective 1-year and 2-year survival rates were 91.3% and 82.2% in the diabetes group and 93.8% and 87.4% in the non-diabetes group. These findings suggest that patients with diabetes on PD have lower survival rates than their counterparts without diabetes. BMI and renal Kt/V values at enrollment were significantly higher in the diabetes group than in the non-diabetes group; however, the older age, higher rate of CVD comorbidity, higher CRP level, and lower HDL cholesterol level at enrollment might be attributed to lower survival rate in the diabetes group.

The diabetes group in our cohort consisted of patients with ESKD caused by diabetic nephropathy and those with diabetes as a comorbid condition. Previous studies of patients on HD have compared those with and without diabetic nephropathy as the primary kidney disease and showed poorer survival in those with diabetic nephropathy^15,16^. Furthermore, The Netherlands Cooperative Study on the Adequacy of Dialysis (NECOSAD) reported that mortality in patients with diabetes as the primary kidney disease was similar to that in patients with diabetes as a comorbid condition^17^. Therefore, diabetes has a strong impact on survival even if it is not the primary cause of ESKD; in our cohort, cardiovascular mortality and new-onset CVD rates were higher in the diabetes group. Patients on PD are exposed to a large amount of glucose absorbed from the dialysate; therefore, continuous exposure to dialysate might worsen glycemic control and cause peritoneal damage in patients with diabetes^18^. Peritoneal absorption of glucose degradation products might enhance formation of advanced glycosylation end products (AGEs), a nonenzymatic reaction between reducing sugars and proteins^19,20^. In the PD population, increased peritoneal membrane transport and protein permeability is more common in patients with diabetes than in those without diabetes, and these characteristics has been shown to be associated with a number of risk factors for mortality and technique failure, including age, racial origin, BMI, burden of comorbid illness, hypoalbuminemia, and elevated inflammatory markers^21–23^. Patients with diabetes on PD may have higher levels of peritoneal AGEs associated with endothelial dysfunction and atherosclerotic CVD^24,25^. However, it was reported that overall survival of patients on PD was significantly higher than that of those on HD with diabetes on incident dialysis, and that the better patient survival on PD was influenced by the degree of glycemic control, that is, an HbA1c level below 8.0%^26^. The present study revealed that 1.5% glucose dialysate and icodextrin dialysate are frequency used in Japan. However, given that the PD dialysate prescription patterns vary from country to country, further studies are needed to clarify whether use of dialysate with a low glucose concentration might affect patient survival.

PD + HD combination therapy has been approved in Japan since 2010 and was used by 18.8% of the patients in our study cohort. Combined therapy is indicated for patients in whom adequate solute clearance (total Kt/V urea >1.7) cannot be maintained with a standard PD prescription and for patients with symptoms of uremia despite a total Kt/V urea >1.7 or a state of persistent fluid overload. It was reported that 50% of patients on combined therapy had a PD duration of more than 8 years^27^. In the present cohort, the duration of PD was significantly longer in the patients receiving combination therapy than in those receiving PD alone (72 months vs. 38 months, P < 0.0001). On the day of HD and on the following day, patients on combination therapy have a PD holiday, that is, they are released from bag exchange for PD^28,29^, which might lead to a decrease in the D/P Cr ratio obtained from the peritoneal equilibration test, suggesting improved functioning of the peritoneal membrane. PD + HD combination therapy is widely used in Japanese patients on PD in view of its known effectiveness for solute clearance and fluid management^30,31^. In one report, there was a decrease in body weight, blood pressure, and serum creatinine, as well as an increase in hemoglobin level, after switching from PD alone to combined therapy, suggesting significant improvement in dialysis and decreased fluid overload^32^. In the present study, all-cause mortality was significantly lower in patients who were receiving combination therapy than in those on PD alone. However, patients selected for PD + HD combination therapy might be different from those in the general PD population, which would influence all-cause mortality and other clinical outcomes. Furthermore, we could not compare the characteristics, including duration of PD and HD, of patients on PD + HD combination therapy in this study. Therefore, further prospective cohort studies are needed to determine whether this combination therapy has a survival benefit, since the patients receiving PD + HD combination therapy and those receiving only PD therapy may constitute different cohorts.

This study has several limitations. First, as in any annual survey or observational cohort study, facility bias could not be excluded. Therefore, the mortality rate and incidence of peritonitis may have varied between centers because of differences in practice and in patient populations. Second, the follow-up duration of 2 years was relatively short, and prevalent patients rather than incident patients were included. Therefore, there are several factors that could vary according to duration of PD, including residual renal function, total Kt/V, and type of dialysate used. Furthermore, early technique failure (within the first 12 months of commencement of PD) is common, and peritonitis is one of the causes of early technique failure. Therefore, we could not detect patients with early technique failure or the exact rate of development of peritonitis. It is not known whether the rates of peritonitis were affected by differences in patient education on how to prevent these complications. Similarly, the effects of physical activity levels, employment status, and socioeconomic factors are unknown. Therefore, prospective studies on whether the outcomes for patients on PD are affected by differences in these factors are required in the future. Finally, glycemic control in patients with diabetes was not assessed because of the small number of patients with data on glycemic indices. Further studies would be needed to assess the important issue of whether the outcomes in diabetes patients on PD would be affected by glycemic control.

In conclusion, older age, longer duration of PD, use of 2.5% glucose dialysate, lower serum albumin level, more severe inflammatory status, comorbid CVD, and higher phosphate level were significant predictors of mortality in Japanese patients on PD. Higher BMI and HDL cholesterol levels were associated with lower mortality risk in these patients. This study identified that all-cause mortality rate is higher in diabetes patients on PD than in their counterparts without diabetes. Further studies are needed to clarify whether PD + HD combination therapy has a survival benefit. The findings of this study underscore the need for further research on the factors that influence patient outcomes to identify alternative interventions that would improve the outlook for patients receiving PD.

## Supplementary information

Supplementary Information
