# Supplementary material for: Predictors of outcomes in patients on peritoneal dialysis: A 2-year nationwide cohort study
Source: Sci Rep. 2019 Mar 8;9:3967. doi: 10.1038/s41598-019-40692-6 (PMC6408436; doi:10.1038/s41598-019-40692-6)
Supplement: Supplementary file 1 — Supplementary Information [file 41598_2019_40692_MOESM1_ESM.pdf]

# **Predictors of outcomes in patients on peritoneal dialysis: A 2-year nationwide cohort study**

Masanori Abe [1,2], Takayuki Hamano [1,3], Junichi Hoshino [1,4], Atsushi Wada [1,5], Shigeru Nakai [1,6], Norio Hanafusa[1,7], Ikuto Masakane [1,8], Kosaku Nitta[1,9], Hidetomo Nakamoto[1,10]

1 The Committee of Renal Data Registry, the Japanese Society for Dialysis Therapy, Tokyo, Japan

2 Division of Nephrology, Hypertension and Endocrinology, Department of Internal Medicine, Nihon University School of Medicine, Tokyo, Japan

3 Department of Inter-Organ Communication Research in Kidney Disease, Osaka University Graduate School of Medicine, Osaka, Japan

4 Nephrology Center, Toranomon Hospital, Tokyo, Japan

5 Department of Nephrology, Kitasaito Hospital, Asahikawa, Japan

6 Department of Clinical Engineering, Fujita Health University, Aichi, Japan

7 Department of Blood Purification, Tokyo Women's Medical University, Tokyo, Japan

8 Yabuki Hospital, Yamagata, Japan

9 Department of Nephrology, Tokyo Women's Medical University, Tokyo, Japan

10 Department of General Internal Medicine, Saitama Medical University, Saitama, Japan

**Supplementary Table 1. Multivariate analysis for variables evaluated as potential predictors of peritonitis in the study population**

| | n (%) | HR | 95% CI | p-value |
|---|---|---|---|---|
| Sex | | | | |
| Male | 5,714 (63.8) | 1.000 | Reference | Reference |
| Female | 3,240 (36.2) | 0.823 | 0.550–1.232 | 0.344 |
| Age | | | | |
| 1-year increase | 8,954 (100) | 1.010 | 0.994–1.026 | 0.203 |
| Duration of PD (years) | | | | |
| <2 | 3,507 (39.2) | 1.000 | Reference | Reference |
| ≥2 <4 | 2,344 (26.2) | 1.011 | 0.658–1.554 | 0.958 |
| ≥4 <6 | 1,422 (15.9) | 0.912 | 0.364–2.287 | 0.845 |
| ≥6 <8 | 761 (8.5) | 0.577 | 0.242–1.375 | 0.215 |
| ≥8 <10 | 414 (4.6) | 0.492 | 0.168–1.436 | 0.194 |
| ≥10 | 506 (5.6) | 0.673 | 0.236–1.918 | 0.459 |
| Diabetes | | | | |
| No | 5,635 (62.9) | 1.000 | Reference | Reference |
| Yes | 3,319 (37.1) | 1.428 | 0.995–2.049 | 0.053 |
| Comorbid CVD | | | | |
| No | 7,399 (84.9) | 1.000 | Reference | Reference |
| Yes | 1,313 (15.1) | 1.992 | 1.302–3.049 | 0.002 |
| Use of antihypertensive medication | | | | |
| No | 1,264 (20.8) | 1.000 | Reference | Reference |
| Yes | 4,802 (79.2) | 1.093 | 0.645–1.852 | 0.741 |
| Hemoglobin | | | | |
| 1 g/dL increase | 6,130 (68.5) | 0.995 | 0.858–1.153 | 0.181 |

| | | | | |
|---|---|---|---|---|
| C-reactive protein | | | | |
| 1-mg/dL increase | 5,344 (59.7) | 1.125 | 0.937–1.350 | 0.221 |
| Body mass index | | | | |
| 1-kg/m$^2$ increase | 5,330 (59.5) | 1.066 | 1.016–1.118 | 0.008 |
| Serum albumin | | | | |
| 1-g/dL increase | 6,092 (68.0) | 1.112 | 0.715–1.728 | 0.626 |
| Residual renal function | | | | |
| Non-anuric | 3,479 (77.2) | 1.000 | Reference | Reference |
| Anuric | 1,026 (22.8) | 1.255 | 0.709–2.222 | 0.434 |
| Total Kt/V | | | | |
| <1.1 | 286 (8.7) | 1.709 | 0.835–3.498 | 0.142 |
| ≥1.1 <1.4 | 326 (9.9) | 0.985 | 0.497–1.953 | 0.967 |
| ≥1.4 <1.7 | 682 (20.7) | 1.000 | Reference | Reference |
| ≥1.7 <2.0 | 853 (25.9) | 0.921 | 0.530–1.599 | 0.771 |
| ≥2.0 | 1,144 (34.8) | 1.246 | 0.746–2.082 | 0.400 |
| Use of automated PD system | | | | |
| No | 3,115 (56.8) | 1.000 | Reference | Reference |
| Yes | 2,366 (43.2) | 1.376 | 0.976–1.938 | 0.067 |
| Use of 2.5% dialysate | | | | |
| No | 3,422 (62.5) | 1.000 | Reference | Reference |
| Yes | 2,056 (37.5) | 1.150 | 0.748–1.767 | 0.522 |
| Use of icodextrin dialysate | | | | |
| No | 3,241 (59.2) | 1.000 | Reference | Reference |
| Yes | 2,237 (40.8) | 0.787 | 0.536–1.155 | 0.221 |
| PD + HD combination therapy | | | | |
| No | 7,275 (81.2) | 1.000 | Reference | Reference |
| Yes | 1,679 (18.8) | 0.898 | 0.493–1.633 | 0.725 |

| | | | | |
|---|---|---|---|---|
| Connecting device | | | | |
| No | 1,699 (31.8) | 1.000 | Reference | Reference |
| Yes | 3,636 (68.2) | 1.885 | 1.249–2.844 | 0.003 |
| Previous peritonitis | | | | |
| No | 4,626 (87.0) | 1.000 | Reference | Reference |
| Yes | 692 (13.0) | 2.222 | 1.382–3.572 | 0.001 |
| Non-HDL cholesterol | | | | |
| 1-mg/dL increase | 3,663 (40.9) | 1.003 | 0.997–1.007 | 0.316 |
| HDL cholesterol | | | | |
| 1-mg/dL increase | 4,264 (47.6) | 0.993 | 0.983-1.003 | 0.218 |
| Calcium | | | | |
| 1-mg/dL increase | 6,175 (69.0) | 1.021 | 0.949-1.096 | 0.574 |
| Phosphate | | | | |
| 1-mg/dL increase | 7,340 (82.0) | 0.982 | 0.842–1.144 | 0.816 |

CI, confidence interval; CVD, cardiovascular disease; HD, hemodialysis; HDL, high-density lipoprotein; HR, hazard ratio; PD, peritoneal dialysis

**Supplementary Figure 1.** a, Comparison of cumulative cardiovascular survival rates between diabetes and non-diabetes patients on peritoneal dialysis. b, Comparison of cumulative infection-related survival rates between diabetes and non-diabetes patients on peritoneal dialysis.

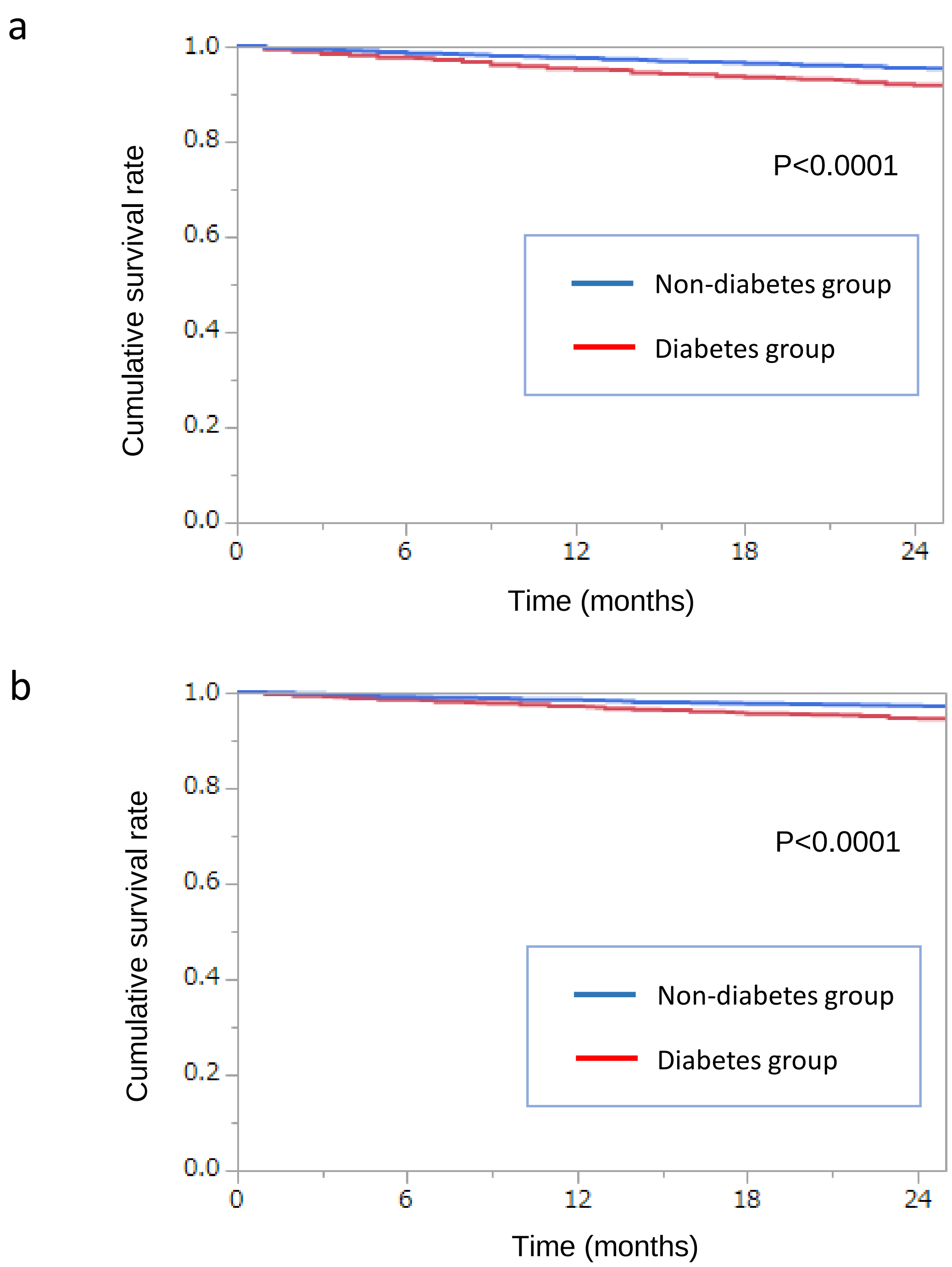
a
1.0
0.8
0.6
0.4
0.2
0.0
Cumulative survival rate
0
6
12
18
24
Time (months)
P<0.0001
Non-diabetes group
Diabetes group
b
1.0
0.8
0.6
0.4
0.2
0.0
Cumulative survival rate
0
6
12
18
24
Time (months)
P<0.0001
Non-diabetes group
Diabetes group
